# Quantifying the probability of pharmacological success to inform compound progression decisions

**DOI:** 10.1371/journal.pone.0240234

**Published:** 2020-10-12

**Authors:** Xuan Zhou, Ole Graff, Chao Chen

**Affiliations:** 1 Clinical Pharmacology Modelling and Simulation, GlaxoSmithKline, Shanghai, China; 2 Discovery Medicine, GlaxoSmithKline, Upper Providence, Pennsylvania, United States of America; 3 Clinical Pharmacology Modelling and Simulation, GlaxoSmithKline, London, United Kingdom; Royal College of Surgeons in Ireland, IRELAND

## Abstract

The Probability of Pharmacology Success, or PoPS, is a powerful metric to inform progression decisions by quantifying a compound’s overall pharmacological strength based on its mechanism. It is defined as the probability that X level of pharmacology is achieved in Y proportion of patients at a safe dose. The importance of adequate drug exposure, target engagement and functional pharmacology for enabling a compound’s efficacy is widely recognized. The PoPS estimates how well these conditions are met by integrating the compound’s pharmacological properties and the target’s modulation needs for the intended indication, in a pharmacometric model that includes the knowledge uncertainty. We use examples to illustrate how it can be used to compare drug candidates under specified benefit and risk conditions, support first-in-human decisions based on exposure limits, advise preclinical lead optimisation, and define clinical-trial populations.

## Introduction

Drug development is a process of attrition: only about 15% of lead compounds approaching preclinical candidate selection advance into clinical trials, [1, 2] and only about 10% of compounds in clinical trials eventually become medicines [3, 4]. For every compound, progression decisions are made along its development program, each primarily based on the efficacy and toxicity data that are available at the time. The soundness of these decisions is vital, for not accidentally abandoning a compound with good potential, and for timely divesting the resource away from a compound that truly has no future.

The understanding of the drug-disease system is in many instances uncertain, particularly for early-stage programs. The pharmacokinetics (PK) and pharmacodynamics (PD) in human are predicted from animal models and *in vitro* experiments. The relevance of the drug target and its pathway to the disease, and consequently the levels of target engagement and pathway pharmacology that are needed for effective intervention, are often speculated using indirect evidence from epidemiology, genetics and animal research. With uncertainty in both what constitutes the success and the extent to which that success can be achieved, estimating a compound’s probability of success is as challenging as it is important. This multi-dimensional ambiguity, combined with human subjectivity, challenges clear and disciplined decision making for compound progression.

The pharmacological prerequisites for a compound to produce efficacy have been explained in terms of adequate attainment of i) effect site exposure to drug concentration, ii) target engagement and iii) pathway pharmacology [5]. Statistics on clinical proof-of-concept showed that only when these conditions are met, a confident progression decision could be made [5]. However, the fragmented nature of the drug-disease system knowledge and the collective uncertainty mean that the expected strength of a drug candidate is inevitably ambiguous. To facilitate objective decision making, we need to be able to objectively and transparently assess the overall pharmacological strength–in terms of the level of pharmacological coverage and the proportion of the patients achieving this level—of an asset for its intended indication, accounting for the uncertainties around both drug properties and intervention needs.

A concept of Probability of Pharmacological Success (PoPS) has been proposed, for quantifying the extent to which the pharmacological prerequisites can be met [6]. It is defined as the probability that X level of pharmacology is achieved in Y proportion of the patient population that the drug is intended for, e.g., the probability that 90% target engagement can be achieved in 80% of patients. In this paper, we use two applications to show how the PoPS concept can be used to assess a compound’s overall pharmacological strength in a single probability term, and to effectively inform the progression decision. The applications further illustrate that, by exploring the impact of the uncertainties in the disease mechanism and in drug attributes, the PoPS assessment can also reveal insight that is useful for steering preclinical lead optimization and identifying suitable clinical-trial population.

### Case background

The indication in question was a rare brain disease; and the drug target was expressed both centrally and peripherally. The activity of the target was known to be elevated in the disease. But there was inconsistency in the mean elevation, estimated from small groups of patients and ranging from 1.5 to 3 folds of the normal level. Data from animals showed evidence of target-related peripheral organ toxicity at high levels of peripheral inhibition. The defining requirement of the pharmacological intervention, on benefit-risk balance, was to normalize the central activity of the target, while preserving necessary peripheral activity.

### Application 1

Two lead compounds were ready for preclinical candidate selection. A decision was required to choose one of them for further preclinical development in preparation for clinical testing. An *in vivo* PK-PD model for central and peripheral inhibition in human was constructed for each compound, including between-subject variability for key parameters. The range of the target activity elevation found in literature and the range of brain-plasma free-drug partitioning among animal species were used as uncertainties for the respective parameters. For each compound, PK-PD data for a large population of patients over a wide dose range were simulated for each set of parameter values. The PoPS, based on this parameter uncertainty, was then calculated according to pre-defined success criteria. The PoPS comparison between the compounds was used to inform the candidate selection decision.

### Application 2

One of the two compounds in *Application 1* clearly promised a much higher PoPS; hence it was chosen for further preclinical development. When the results from the animal toxicology experiments became available, safety concerns required a minimum of 10-fold exposure margin, defined as the ratio for the area under the daily plasma drug concentration curve (AUC) between the No-Observed-Adverse-Effect-Level (NOAEL) dose in the toxicology study and the clinical dose. A decision whether to start the first-in-human trial (FiH) for this compound called for an assessment of the overall probability of success of the compound. This was a judgement based on the likelihood of adequate pharmacology that could be expected at a safe dose. In this context, the highest PoPS within the safe exposure range was considered as the compound probability of success (PoS), to support the decision whether to progress the compound into FiH.

## Materials and methods

### Pharmacokinetic-pharmacodynamic model

Both compounds were predicted to have first-order absorption, first-order elimination and one compartment distribution. Oral clearance, as the ratio of intravenous clearance to oral bioavailability (CL/F), was predicted from PK in multiple animal species. Central and peripheral inhibitions in the *i*th individual (*INH*_ci_ and *INH*_pi_) were described by simple *E*_max_ models shown in Eq 1 and Eq 2, where *I*_maxi_, *IC*_50i_, *k*_p,uui_ and *C*_avg_ssi_ represented the individual maximum inhibition, free concentration causing 50% of the maximum inhibition, free compound brain-plasma partitioning coefficient, and steady-state average plasma free concentration. For the population geometric mean values, the *IC*_50_ was derived from *in vitro* human cell lines, the *I*_max_ was assumed to be 100% as observed *in vitro*, the *k*_p,uu_ was based on *in vitro* measurement from animals, and the *C*_avg_ss_ was calculated as daily dose divided by CL/F and multiplied by free fraction of the drug in human plasma. The geometric mean values for CL/F, Kpuu and pIC50 were 6.27 L/h, 0.45–0.75 and 8.3 for Compound A; and 18.8 L/h, 0.35–0.50 and 8.2 for Compound B. Parameters (CL/F)_i_, *k*_p,uui_ and *IC*_50i_ were assumed to follow log-normal distribution, with a moderate 30% between-subject variability implemented by using the parameter standard deviation of 0.3 on the log scale.

$$INH_{ci}(\%)=\frac{I_{maxi}C_{avg\_ssi}k_{p,uui}}{IC_{50i}+C_{avg\_ssi}k_{p,uui}}\cdot 100$$ (1)

$$INH_{pi}(\%)=\frac{I_{maxi}C_{avg\_ssi}}{IC_{50i}+C_{avg\_ssi}}\cdot 100$$ (2)

To achieve enough central inhibition, Subject i, with target activity elevation of *Fe*_*i*,_ would need an *INH*_*ci*_ of at least 1-1/*Fe*_*i*_. To preserve necessary peripheral activity, the same subject should have an *INH*_*pi*_ of no greater than 1–0.1/*Fe*_*i*_.

### Simulation setup

Subject-level data (N = 1000) of inhibition of central and peripheral target activity for 1000 trials were simulated according to the above inhibition functions. Because the treatment success was hypothesized on the needs to normalize the central target activity while preserving the required peripheral target activity, the extent of the activity elevation (*F*_e_) in patients and the ability of the drug to distribute into the brain (*k*_p,uu_) were the key determinants. To account for the uncertainty in these parameters, we applied uniform distribution over the credible ranges of the prior knowledge on population mean values: *F*_e_ of 1.5–3.0 as found in the literature, and *k*_p,uu_ of 0.45–0.75 for Compound A and 0.35–0.50 for Compound B, both measured in multiple animal species. Both *F*_e_ and *k*_p,uu_ parameters were assumed to follow log-normal distribution, with a moderate 30% between-subject variability. Data from each simulated trial then reflected a (unique) combination of the population mean values of these two parameters, as well as the assigned (30%) between-subject variability.

### Estimation of PoPS in application 1

In each of the 1000 simulated trials, the patients could be categorized into four groups according to the drug effect on central and peripheral target activities: Group A for those with sufficient central inhibition (normalized activity) and sufficient (at least 10%) peripheral preservation–the group with desired benefit-risk profile; Group B for those with insufficient central inhibition but sufficient peripheral preservation; Group C for those with sufficient central inhibition but insufficient peripheral preservation; and Group D for those with insufficient central inhibition and insufficient peripheral preservation–a group absent in the simulated conditions. Success criteria for a clinical trial were set as at least 80% patients in Group A (the desirable group), and less than 5% patients in Group C+D (the group with safety risk). The proportion of the trials meeting the criteria was calculated as the success rate, or PoPS. The optimal doses for the two compounds were identified at the highest PoPS. We examined the impact of different success criteria on PoPS and the optimal dose, by varying the requirements for Group A and Group C+D. In addition, the impact of *F*_e_ and *k*_p,uu_ on the sizes of Group A and Group C+D was also investigated.

### Estimation of compound PoS in application 2

For Compound A, a NOAEL dose was identified in animal toxicology experiments; it was associated with a steady-state daily AUC of 38.4 μg.h/mL. Safety concerns required the clinical exposure to have a minimum of 10-fold safety margin, defined as the AUC ratio between the NOAEL and the clinical dose. Therefore, we examined the PoPS as a function of the safety margin. We also explored the size of Group A, in the event of *in vivo IC*_50_ being either higher or lower than the *in vitro* value used in the model.

In the work presented here, all simulations and data analyses were performed by using R and the Simulx function of the R package mlxR (Lixoft®, Orsay, France).

## Results

### Estimation of PoPS in application 1

**Fig 1:**
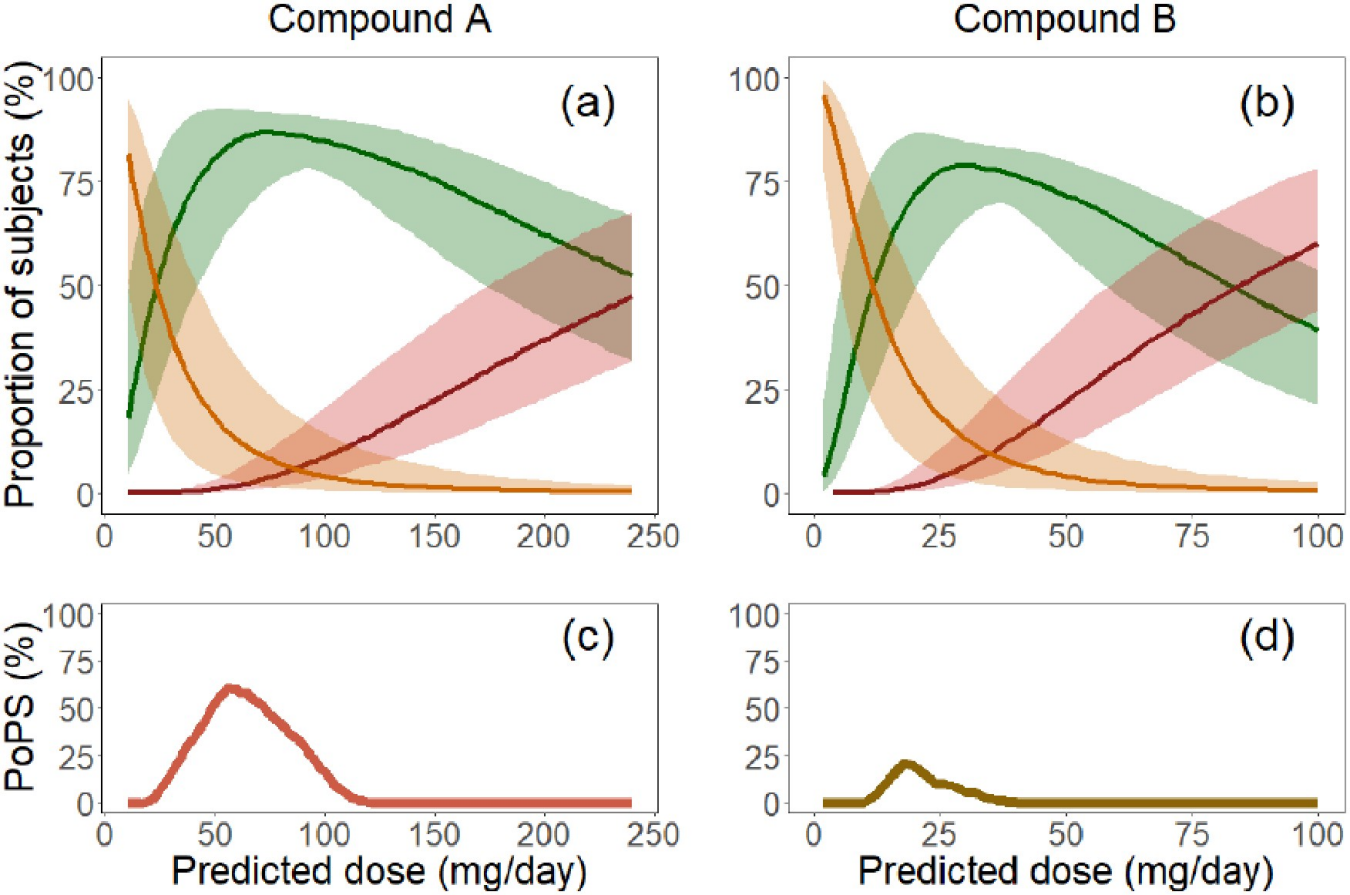
Estimation of probability of pharmacological success (PoPS) for Compound A (left) and Compound B (right). Upper panel: proportions of patients in Group A (Green), Group B (Orange) and Group C+D (Red); the lines are the medians of 1000 scenario simulations; the bands are the 90% prediction intervals from these simulations. Lower panel: PoPS calculated based on criteria: ≥80% patients in Group A and <5% patients in Group C+D.

For Compound A, the highest mean size of Group A for the 1000 simulations was 87%, at the dose of 74 mg/day; for Compound B, this was 79%, at 30 mg/day (Fig 1(a) and 1(b)). However, at these doses, the sizes of Group C+D were 8.4% and 13%, respectively, exceeding the 5% limit of the success criteria. The maximal PoPS was 61% for Compound A (at 56 mg/day) or 21% for Compound B (at 18 mg/day) (Fig 1(c) and 1(d)). Given the clearly higher PoPS, Compound A was selected for further development.

**Fig 2:**
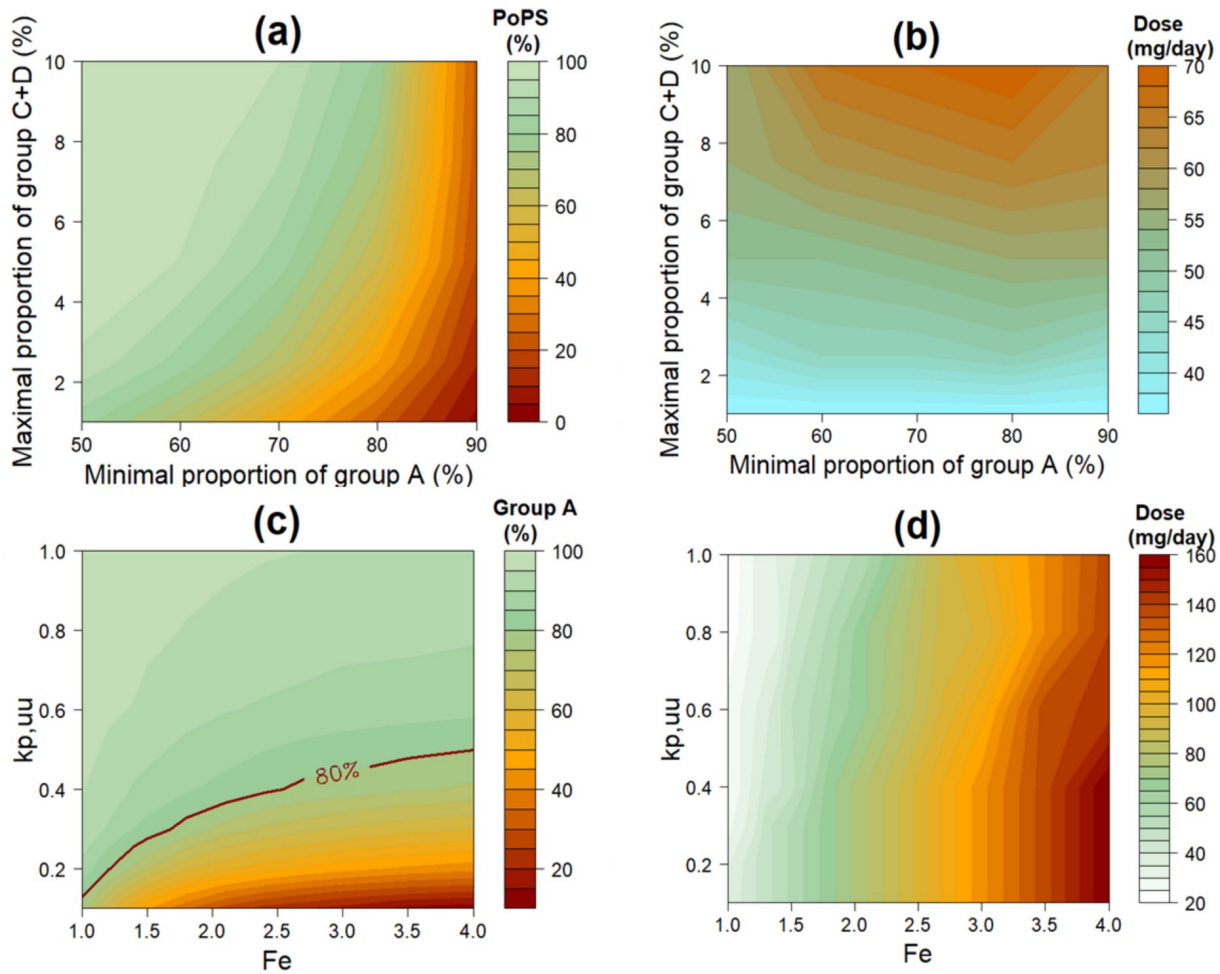
The impact of success criteria and uncertainty in key parameters (target activity elevation Fe and brain penetration kp,uu). (a) the maximal Probability of Pharmacological Success (PoPS) (%) of Compound A based on different success criteria; (b) Compound A dose for the maximal PoPS; (c) maximal proportion of patients in Group A, conditioned on < 5% of patients in Group C+D; (d) Compound A dose for the maximal proportion of patients in Group A.

To assess the impact of success criteria setting, Fig 2(a) shows the maximal PoPS for Compound A under various requirements for the sizes of Group A and Group C+D. The PoPS would increase significantly if larger Group C+D or smaller Group A were allowed. For instance, if Group A were decrease to 60% and Group C+D increase to 10%, Compound A would achieve 99% PoPS. In addition, Fig 2(b) showed a higher dose would be required to achieve the maximal PoPS if a larger Group C+D were allowed.

The impact of parameter uncertainty was also examined. Fig 2(c) shows the Group A size (conditioned on Group C+D <5%) for any combination of plausible values of brain-plasma portioning coefficient (*k*_p,uu_), and target elevation (*F*_e_). Group A size would increase significantly with increasing *k*_p,uu_ and decreasing *F*_e._ This plot also tells us that a necessary Group A size is only achievable by the combination of the parameter values in the area above the line depicting that Group A size. For example, when we needed Group A to be 80% of patients, a compound of *k*_p,uu_ = 0.3 could treat a population with mean *F*_e_ up to 1.5. To treat a population with *F*_e_ up to 2.5 would require a compound with *k*_p,uu_ = 0.4. Besides, *F*_e_ had a major effect on dose requirement: for a given value of *k*_p,uu_, every 0.5-fold increase in *F*_e_ meant the dose of Compound A to be about 20 mg/day higher, to achieve the maximum Group A size (Fig 2(d)).

### Estimation of PoS in application 2

**Fig 3:**
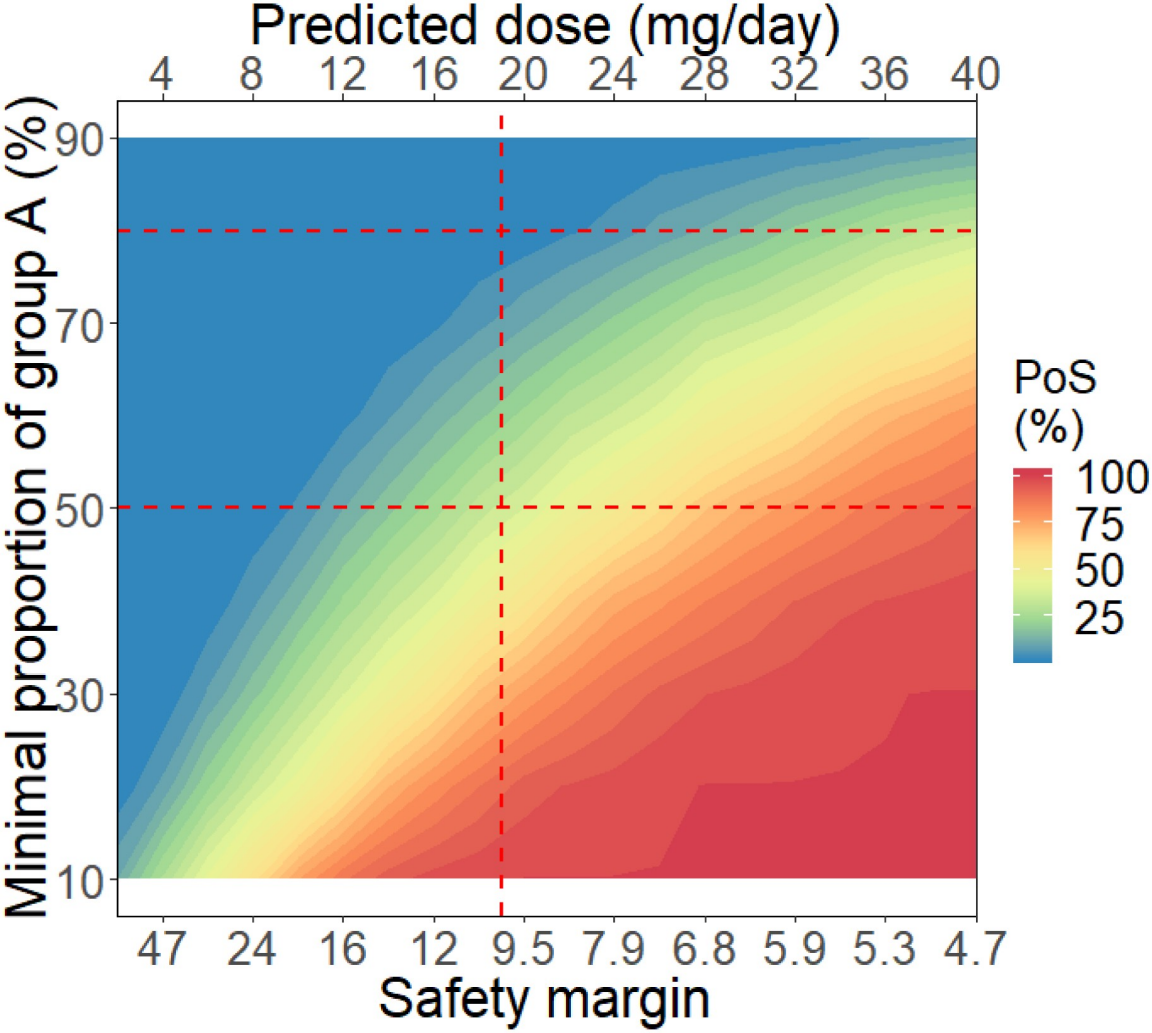
The Probability of Pharmacological Success (PoPS) (%) for having a minimal proportion of patients in Group A (conditioned on <5% patients in Group C+D) as a function of exposure safety margin, or the predicted dose. The horizontal lines represent the requirement of at least 50% and 80% patients in Group A. The vertical line marks 19 mg/day dose that’s predicted to produce a 10-fold exposure safety margin.

The 56 mg/kg dose of Compound A for its maximal PoPS of 61% had a safety margin of 3.4 folds in relation to the NOAEL AUC. The highest dose for the required 10-fold margin would be 19 mg/day. Fig 3 shows that under the agreed success criterion of having ≥80% of patients in Group A, the PoS would be just below 1%. The same figure also shows that under a more relaxed criterion of having ≥50% patients in Group A, the same dose of 19 mg/day could achieve a far higher PoS of 43%.

**Fig 4:**
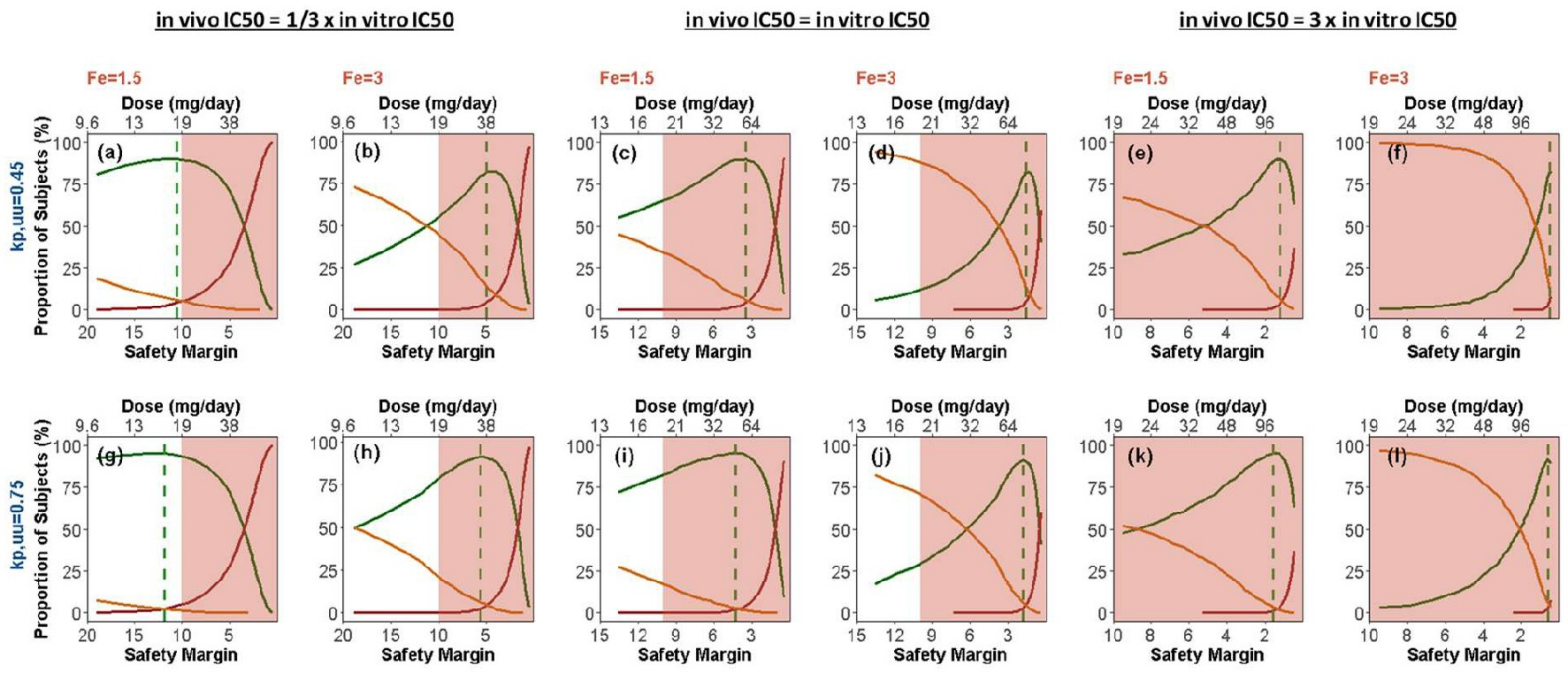
The impact of *in vivo* potency (IC50), brain penetration (kp,uu) and elevation of target activity (Fe) on the pharmacological response. The green, orange and red curves represent the proportions of patients in Groups A, B and C+D, respectively. The vertical lines mark the “optimal dose”, effectively the lower of 1) the dose maximizing patients in Group A and 2) the dose limiting patients in Group C+D to 5% of the population. The shaded areas represent the doses which will generate less than 10-fold safety margin.

In the simulations for PoPS estimation in Application 1, we simply used the *in vitro IC*_50_ measured from patient cell line, because the drug potency had no effect on the maximal PoPS when there was no exposure limit. However, the *in vivo IC*_50_ became a critical parameter when toxicology finding suggested the exposure limit. Therefore, we further assessed the impact of the uncertainty in *IC*_50_, in addition to the impact of uncertainties in *k*_p,uu_ and *F*_e_, as shown in Fig 4. If the *in vivo IC*_50_ were the same as the *in vitro* value, the best-case for Group A size would be near 80% (Fig 4(i)), while the worst case would be only 10% (Fig 4(d)). However, if the *in vivo IC*_50_ were 3-fold lower, Group A size would be more than 90% at best (Fig 4(g)) and still 55% at worst (Fig 4(b)). In contrast, if the *in vivo IC*_50_ were 3 folds of the *in vitro* value, Group A size would be less than 50% at best (Fig 4(k)).

## Discussion

### Enhancing decision quality considering fragmented knowledge and multiple uncertainties

Confident decision making during the early stage of a drug development program requires a mechanism to combine the understanding of drug properties and disease intervention needs, and to address the ambiguity in this understanding [7]. The PoPS concept uses a drug-disease model to provide a structure to integrate the otherwise disjointed knowledge in a coherent way, to estimate the probability for adequate pharmacological coverage, considering both the level of the coverage and the proportion of the patients to be covered. This estimation reflects the overall strength of the drug for its indication, accounting for both the uncertainty in the knowledge and the variability among patients; it can therefore serve as a basis for the asset progression decision.

Our case study shows its application to an endpoint that can be considered as target binding or proximal pharmacology. Early knowledge suggested a two-fold elevation of the target activity in the disease, and *in vivo* test revealed the drug effect as a simple *E*_max_ model. This implied that a central-peripheral free drug partitioning ratio as low as 0.1 would allow both central pharmacology (50% inhibition) and peripheral safety (10% preservation). Before PoPS estimation, the two drugs approaching candidate selection could not be clearly differentiated: simulations showed that the proportion of patients in the group achieving the required central inhibition and peripheral preservation (Group A) would be similar and both high at their respective optimal doses (Fig 1(a) and 1(b)), with Drug A being only marginally better. However, The PoPS values showed that when the requirements for both efficacy (>/ = 80% patients in Group A) and safety (< 5% patients in Group C+D) were considered, Drug A was clearly the winner (Fig 1(c) and 1(d)).

Unlike a confirmatory trial with clearly defined (single) efficacy endpoint for calculating the PoS, the FiH trial is the initial clinical exploration of PK, PD, safety and tolerability in a small number of subjects. Therefore, specifying the success criteria for an asset at this stage could be complicated. Our approach was to apply the PoPS concept according to the simple fundamental principal: a drug may be a viable medicine only if a sufficiently active dose is also safe. We defined the PoS in PoPS terms as the probability that X level of pharmacology in Y proportion of patients can be achieved at any dose that would produce a mean exposure within the limit considered safe based on non-clinical findings. When we needed at least 80% patients with the target central/peripheral inhibitions, the PoS of < 1% in this context provided clarity for the decision not to progress Drug A to FiH (Fig 3).

The concept of PoPS presented here bears some resemblance with the concept of probability of trial success, or assurance [8–10], that is increasingly recognized as an important metrics for assessing the required sample size in clinical trial design. Like the study power, the assurance depends on the assumed drug effect, the trial features, and the analysis method. While the power is the probability of detecting a specific effect, the assurance is the probability of detecting an effect that is modelled on a likely distribution of the potential effects. Hence the assurance is more robust and less restrictive than the power. However, there are several differences between the PoPS and trial assurance. An obvious distinction is that the PoPS is used to assess the strength of a single treatment, of monotherapy or even combination therapy, as opposed to assurance which is usually calculated for a comparison of two treatments. While the PoPS focuses solely on the pharmacological strength of the drug to aid progression decisions, the assurance incorporates the trial operational and analytical factors to aid trial design and analysis considerations. The PoPS usually requires assumption in the necessary level of pharmacological intervention; whereas assurance can be based on the observed drug effect and its uncertainty from previous trials to weigh the power values.

### Revealing insight to steer pre-clinical discovery and clinical development directions

In addition to integrating drug-disease knowledge and its uncertainty to assist decisions on candidate comparison and compound progression, the PoPS estimation and the associated graphics are also useful tools for identifying key knowledge gaps. For example, Fig 2(c) quantifies the importance of the *k*_p,uu_ value for the potential therapeutic success and for patient population selection. This highlights the needs for early understanding of *k*_p,uu_ in human brain tissue, instead of solely relying on animal data. Another example in our case study was the realization of the importance of *IC*_50_ value. The PoPS was calculated using the *IC*_50_ value derived from *in vitro* cell lines. A competitor program later showed moderate discrepancy between the cell-line and *ex vivo* values for its asset, and consistency between *ex vivo* and *in vivo* values estimated from FiH. This prompted us to explore the impact of *IC*_50_ value on Group A size (Fig 4); and the impact turned out to be significant in the context of safe exposure constraint. This finding also stressed the importance of early development of an *ex vivo* assay that better mimics the *in vivo* environment.

The PoPS concept can be a mechanism for assessing the impact of drug- disease system parameters on the potential success of an asset. In the case study, the two key determinants for the size of Group A, the group with desired benefit-risk profile, were drug property *k*_p,uu_ and disease condition *F*_e_. Fig 2(c) shows the maximal size of Group A for any value combination for these two parameters; it defines a viability zone below which the success criterion of having 80% of patients in Group A would not be possible. The plot tells us that an intended patient population whose target elevation, determined either by genotype or phenotype, would need a drug with a *k*_p,uu_ above a certain value; conversely, a drug with a known *k*_p,uu_ would be more effective in lowering the target activity in one sub-population of patients than another. These are useful insights to guide both lead optimization in drug discovery and patient selection in clinical trials. Similarly, Fig 2(d) shows the dependency of the optimal dose (for maximal proportion of successfully treated patients) on the values of *k*_p,uu_ and *F*_e_. This plot also shows that if the daily dose is limited by safety (e.g., gastrointestinal tolerability, liver toxicity or toxic impurity) or cost concerns, the treatment potential would be limited for patients with higher elevation of target activity.

### Practical considerations for implementing the PoPS approach

The PoPS can be applied situationally to a PK or PD endpoint that is considered important for achieving efficacy: drug exposure such as steady state average concentration, maximal concentration or minimal concentration; receptor occupancy; proximal or distal pathway pharmacology; or time above a certain cut-off value of exposure, target binding or pathway pharmacology.

Important for the approach is to identify the key parameters whose uncertainties would impact the probability in question. In our candidate selection example, key to the PoPS was normalizing the target’s central activity and preserving its peripheral activity. Therefore, the relevant parameters were the extent of central elevation to be normalized, and the drug’s brain penetration: the higher the elevation or the lower the penetration, the lower the success. Given a pair of mean elevation and penetration values (and all the between-subject variabilities), the same PoPS would be expected regardless of the mean IC50 or CL/F. A different mean IC50 or CL/F just meant a different exposure or dose requirement (for that same PoPS). In our FiH example, a different mean IC50 could shift the same PoPS to an exposure that was no longer acceptable considering the toxicology findings; therefore, despite clear evidence that the IC50 from the PD assay was highly reproducible, we explored the impact of hypothetical change in IC50 on the proportion of patients achieving adequate pharmacology. Conceivably, a full PoPS re-estimation could have been conducted at this stage. It was not done because the evidence for uncertainty in IC50 was weak.

Once the key parameters are identified, the uncertainty values should be based on the understanding of the disease (in our case the range of the mean elevation of target activity reported in several small studies) and the drug PK-PD (in our case the range of brain-blood free concentration ratio from multiple animal species). The between-subject variability should be based on the PK-PD nature and non-clinical findings of the drug. For PK, drugs with high hepatic extraction ratio or poor oral absorption tend to have more variable concentration. For PD, an ex vivo assay in a challenged condition tends to be more variable between subjects and between occasions, compared to an assay without any challenge. Another example of situational consideration is for anti-bacterial treatments, where the PD variability can be based on the susceptibility distribution of clinical isolates of relevant pathogen strains.

The model that is used for PoPS estimation should be formulated as close to the indication population as our knowledge and data allow. At each development decision point, e.g., from preclinical candidate selection to clinical proof of concept, the quality of data is different in terms of their indication relevance. Consequently, the level of uncertainty and the requirement for assumption are also different. To this end, before clinical data–from patients or even healthy subjects–become available, translational PK-PD modelling using good-quality data from relevant animal disease models can aid the understanding of the required clinically-relevant level of pharmacological response. When a compound progresses along its development pathway, more patient-relevant data become available and less assumption are required.

It should be noted that the PoPS approach does not necessarily address translational uncertainty–whether the pharmacology observed in an *in vitro* or animal model can make a meaningful difference in the disease state in patients. This translational risk, common for drugs with unproven mechanism, can only be reduced by better mechanistic understanding of the disease biology, the importance of the intervention pathway, and the consequence of the drug-target interaction on the pathway. This is the area where quantitative system biology and system pharmacology have the potential to impact. When using in vitro and animal data for estimating the PoPS, the differences between in vitro and *in vivo* settings, and between animals and humans, should be clearly identified and quantitatively captured in the translational model.

Another important point is how to define success criteria. In our example of candidate selection, the criteria were “>/ = 80% of patients achieving normalized central target activity while preserving >/ = 10% peripheral target activity; and at the same time, the total number of patients with < 10% peripheral target activity being < 5%”. These values were admittedly arbitrary; they were chosen by consensus among biologists, toxicologists and clinicians on the project team based on the collective understanding of the disease in the brain, the nature of the peripheral toxicity, and the benefit-risk ratio for the indication. Another situational consideration is around the indication requirement: a compound aimed to improve on existing treatment may require a stricter success criterion than one intended for an indication with high unmet medical needs. To ensure the criteria are rational, the success criteria should be chosen before the PoPS is estimated and reported. On the other hand, plots like Fig 2(a) and 2(b) allow the assessment of the impact of alternative success criteria on the maximal PoPS and on the corresponding optimal dose. They are useful tools to facilitate project team discussions as well as funding decisions by governance boards.

A related aspect is the success probability that is acceptable for a positive decision. In our candidate selection example, one compound showed a much higher probability than the other; the clear difference meant an easy choice. In a situation of a small difference between compounds, other factors such as compound half-life, drug interaction potential, chemical synthesis or formulation development may need to be considered. In our FiH example, the PoS within the safe exposure limit was so low, it was also an easy termination decision. In a situation of a progression decision for a single compound with moderate probability, there are two elements to consider. Firstly, whether any perceived probability level of pharmacological success can indeed lead to (clinically relevant) efficacy at all is a translational question generic for drugs with unproven mechanism, which needs to be answered with eventual efficacy data in patient trials. Secondly, assuming the pharmacological success criteria are meaningful for the eventual clinical efficacy, a lower PoPS reflects a higher risk of achieving the efficacy. Therefore, it is also a business decision to be made. Factors such as the level of unmet medical needs, the organization’s tolerance of investment risk or the competitive landscape would need to be considered.

Decisions must be made at risk whether to invest resources into progressing a compound. They are typically faced with a complex situation of many uncertainties including the level of required pharmacological intervention for the intended indication, as well as the drug’s PK, PD and safety profiles. Traditionally, these decisions are made intuitively, when the strengths and weaknesses counteract against each other, and with further complications of knowledge gaps. Depending on an individual’s selective awareness, preference and bias, these decisions can be highly subjective and arbitrary. The purpose of PoPS is to estimate the pharmacological strength of the compound in the eventual patient population when, by nature, such patient data are not yet available. Therefore, predictions are necessarily based on knowledge gained from in vitro experiments and animal models. We view PoPS as a mechanism to integrate a compound’s relevant strengths and weaknesses, to identify the knowledge gaps, and to put reasonable level of uncertainties to necessary assumptions. It is therefore a decision enhancer—a framework to help make the decisions more rational and transparent. By nature of a probabilistic tool, its validity assessment would require many retrospective cases. Gathering enough cases with clear pharmacological data in patients would require considerable time and a large portfolio of compounds, given the fact that most development programs are terminated early due various unforeseen circumstances including strategy shift. However, through identifying the required level of drug intervention to base the success criteria on, the PoPS estimation serves as a mechanism to ensure there is adequate understanding of how the pharmacological assay can relate to clinical efficacy.

## Conclusions

Adequate PK and PD coverage is essential for a drug’s therapeutic success. The model-based estimation of PoPS is a powerful and versatile approach for quantifying this coverage considering the uncertainties in the drug-disease system. It can be used to effectively integrate multi-source data and to transparently consolidate the relevant uncertainties in both drug properties and intervention needs into a single probability term, providing clarity for making rational at-risk decisions in drug development. It can also potentially generate insight to help steer discovery efforts and identify patient populations for clinical trials.
